# Change in Plant-Based Diet Quality Is Associated with Changes in Plasma Adiposity-Associated Biomarker Concentrations in Women

**DOI:** 10.1093/jn/nxy301

**Published:** 2019-03-30

**Authors:** Megu Y Baden, Ambika Satija, Frank B Hu, Tianyi Huang

**Affiliations:** 1 Departments of Nutrition, Harvard TH Chan School of Public Health, Boston, MA; 2 Departments of Epidemiology, Harvard TH Chan School of Public Health, Boston, MA; 3 Channing Division of Network Medicine, Department of Medicine, Brigham and Women's Hospital, Boston, MA

**Keywords:** plant-based diet index, biomarker, healthful plant-based diet, obesity, inflammation

## Abstract

**Background:**

A healthful plant-based diet is associated with lower risk of cardiometabolic diseases. However, it is still unclear whether such benefits are due to its favorable effects on adiposity-associated biomarkers.

**Objective:**

We investigated the associations between biomarkers and 3 plant-based diet indices: an overall plant-based diet index (PDI); a healthful plant-based diet index (hPDI); and an unhealthful plant-based diet index (uPDI).

**Methods:**

In the Nurses’ Health Study II, 831 women [baseline mean age: 45 y; body mass index (BMI, kg/m^2^): 24.6] were randomly selected from those who provided 2 blood samples in 1996–1999 and 2010–2011 to measure plasma concentrations of adiponectin, leptin, soluble leptin receptor (sOB-R), insulin, retinol-binding protein-4, high-sensitivity C-reactive protein (hsCRP), and interleukin-6 (IL-6). Plant-based diet indices were derived from semiquantitative food frequency questionnaires assessed at each blood collection. Linear mixed models were used to evaluate cross-sectional associations, and general linear models were used to evaluate longitudinal associations.

**Results:**

In cross-sectional analyses with multivariable adjustment including BMI, higher hPDI was associated with lower concentrations of leptin, insulin, and hsCRP, and higher adiponectin and sOB-R concentrations (biomarker differences per 10-point higher hPDI: −7.2%, −10.0%, −13.6%, 3.0%, and 1.9%, respectively; *P* ≤ 0.025). A higher uPDI was associated with higher concentrations of leptin and insulin (4.4% and 4.8%, respectively; *P* ≤ 0.048). In longitudinal analyses with multivariable adjustment including weight change, an increase in hPDI (improved plant-based diet quality) was inversely associated with changes in leptin and hsCRP (biomarker changes per 10-point hPDI increase: −7.7% and −17.8%, respectively; *P* ≤ 0.005), whereas an increase in uPDI (worsened plant-based diet quality) was positively associated with changes in leptin, hsCRP, and IL-6 (10.1%, 13.5%, and 12.4%, respectively; *P* ≤ 0.021).

**Conclusions:**

Adherence to a healthful plant-based diet is associated with favorable long-term changes in adiposity-associated biomarker concentrations in women.

## Introduction

Obesity has become a worldwide problem, and is a major risk factor for type 2 diabetes (T2D), coronary heart disease (CHD), stroke, and cancer (1–4). Excess visceral fat induces dysregulation of biomarkers that play crucial roles in promoting inflammation and insulin resistance. For example, leptin, adiponectin, and retinol-binding protein-4 (RBP-4) are adipokines secreted from adipose tissues. Positively associated with obesity, leptin acts as a proinflammatory cytokine and its biological activity is partly regulated by soluble leptin receptor (sOB-R), the primary leptin-binding protein in circulation (5, 6). By contrast, adiponectin is inversely associated with obesity, and reduces inflammation and insulin resistance (7–9). RBP-4 is another adipokine that transports retinol (vitamin A) and is elevated among individuals with insulin resistance (10). In addition, the accumulation of abdominal fat has been associated with increasing concentrations of other inflammatory biomarkers such as C-reactive protein (CRP) and IL-6 (11, 12).

Diet is a modifiable factor of obesity and obesity-related diseases, and there has been growing interest in plant-based diets, which have been associated with a lower risk of CHD, T2D, and other cardiometabolic disease (13–17). However, previous studies on plant-based or vegetarian diets are limited because they did not differentiate the quality of plant-based foods. For example, certain plant-based foods, such as refined grains and sugar-sweetened beverages, are associated with higher cardiometabolic risk (18–20). To address this, we recently developed 3 plant-based diet indices that reflect the quality of plant-based foods, including an overall plant-based diet index (PDI), a healthful plant-based diet index (hPDI), and an unhealthful plant-based diet index (uPDI). The hPDI captures a high-quality plant-based diet rich in whole grains, fruits, vegetables, and nuts and low in fruit juices, refined grains, and sweets, whereas uPDI represents the opposite. We previously reported that hPDI and uPDI were differentially associated with the risk of T2D and CHD (21, 22). Nevertheless, to what extent a healthful plant-based diet is associated with adiposity-associated biomarkers remained unclear. To our knowledge, no study to date has evaluated the long-term associations between changes in plant-based diet and changes in biomarkers related to cardiometabolic diseases.

Therefore, in this study, we followed healthy US women for an average of 13 y to elucidate whether PDI, hPDI, and uPDI were associated with several biomarkers predictive of cardiometabolic diseases. We hypothesized that improvement in hPDI could be associated with favorable changes in circulating concentrations of adiponectin, leptin, sOB-R, insulin, RBP-4, hsCRP, and IL-6.

## Methods

### Study population

The Nurses’ Health Study II was established in 1989 among 116,686 US female registered nurses aged 25–42 y. All women completed a baseline questionnaire, and lifestyle factors and medical history were updated every 2 y. The first blood sample was provided by 29,611 women in 1996–1999, and the second blood sample was provided by 15,982 women in 2008–2011. Of these, 850 women were randomly selected from those who provided 2 fasting (≥8 h before blood collection) blood samples in both 1996–1999 and 2010–2011 (23). The current analysis included 831 women who were free from cancer, cardiovascular disease, and T2D, and had dietary data available at both blood collections. The study protocol was approved by the institutional review boards of the Harvard TH Chan School of Public Health and Brigham and Women's Hospital. The completion of self-administered questionnaires was considered to imply informed consent.

### Diet assessment

Beginning in 1991, dietary data were collected every 4 y using a semiquantitative FFQ. Participants reported how often, on average, they had consumed defined portions of the 130 food items over the previous year using 9 response categories, ranging from “never or less than once/month” to “≥ six times/day.” The reliability and validity of the FFQ have been described elsewhere (24, 25). We have previously reported the method to derive the 3 plant-based diet indices from FFQs that differentiate the quality of plant-based foods (21, 22). Briefly, we first created 18 food groups based on nutrients and culinary similarities within the larger categories of healthy plant foods (whole grains, fruits, vegetables, nuts, legumes, vegetable oils, tea/coffee); less healthy plant foods (fruit juices, refined grains, potatoes, sugar-sweetened beverages, sweets/desserts); and animal foods (animal fat, dairy, eggs, fish/seafood, meat, miscellaneous animal-based foods). Food groups were ranked into quintiles, and given positive or reverse scores. For creating a PDI, foods in both plant food groups were given positive scores, and foods in the animal food group were given reverse scores. For the hPDI, foods in the healthy plant food group were given positive scores, and foods in the less healthy plant food group and the animal food group were given reverse scores. For the uPDI, foods in the less healthy plant food group were given positive scores, and foods in the healthy plant food group and the animal food group were given reverse scores. Finally, the 18 food group scores were summed to obtain 3 plant-based diet indices, with a theoretical range of 18 to 90. The FFQ in either 1995 or 1999 (whichever was closer to the first blood measurement) was used as the baseline dietary assessment, and the FFQ in 2011 was used as the follow-up assessment (the year close to the second blood measurement).

### Plasma biomarker measurements

Plasma samples were stored in the vapor phase of liquid nitrogen (LN2) freezers with LN2-rated gasketed screw tops and labels since collection. Plasma biomarker concentrations were measured in the Clinical Chemistry Laboratory at Boston Children's Hospital. Leptin, sOB-R, RBP-4, and IL-6 were measured by an ultrasensitive ELISA assay (R&D Systems). Total adiponectin was assayed with a quantitative monoclonal sandwich ELISA (Alpco Diagnostics). Insulin was determined by an electrochemiluminescence immunoassay using the Roche E modular system (Roche Diagnosis). High-sensitivity C-reactive protein (hsCRP) was measured by an immunoturbidimetric assay (Denka Seiken). The mean interassay coefficients of variation (CVs) were 4.9% for leptin, 11.1% for sOB-R, 10.1% for adiponectin, 6.4% for insulin, 10.3% for RBP-4, 1.5% for hsCRP, and 10.8% for IL-6 (23). The free leptin index was calculated as the ratio of leptin to sOB-R to reflect the unbound leptin concentration in circulation (26).

### Covariate assessments

Date of birth and height were collected at baseline. Biennial follow-up questionnaires updated information on body weight, disease diagnoses, menopausal status, postmenopausal hormone use, and several lifestyle factors including physical activity, alcohol intake, and smoking status. Hypertension and hypercholesterolemia were self-reported by responding to the questions whether the participant had clinician-diagnosed “high blood pressure” or “elevated cholesterol.” We used the covariate information collected concurrently along with the baseline and follow-up FFQs (i.e., either 1995 or 1999 for baseline and 2011 for follow-up).

### Statistical analysis

All biomarker concentrations and the free leptin index were log-transformed to normalize distributions and then outliers were excluded. Participants were divided into 5 groups according to the quintiles of PDI scores in the cross-sectional analysis, and the quintiles of changes in PDI scores in the longitudinal analysis. We first evaluated the age-standardized distribution of participant characteristics at each blood collection, and the differences between the 2 collections were compared with the use of paired *t* tests for continuous variables and McNemar's tests for binary variables. Linear mixed-effect models were used to examine the cross-sectional associations of PDI, hPDI, and uPDI with biomarker concentrations, with an unstructured covariance matrix specified to account for within-person correlations between the 2 time points. Least squares geometric mean concentrations were estimated for each biomarker according to quintiles of the 3 PDIs, and adjusted for time-varying covariates corresponding to each blood collection, including age (continuous), total energy intake (kcal; in quintiles), alcohol intake (g/day; in quintiles), smoking status (current, past, never), physical activity (metabolic equivalent task hours per week, MET-h/week; in quintiles), menopausal status (premenopausal, postmenopausal), postmenopausal hormone use (current, past, never), hypertension (yes, no), and hypercholesterolemia (yes, no). An indicator variable was also included to differentiate between the first and second blood collection. To elucidate whether associations were independent of obesity, further adjustment for BMI (<20.0, 20.0–24.9, 25.0–29.9, ≥30.0 kg/m^2^) assessed at each sample collection was made in a separate model. Linear trends were tested by using the median score of each quintile of an index as a continuous variable, and the percentage difference in biomarker concentrations was estimated for every 10-point difference of each index.

In the longitudinal analysis, multivariate general linear regression models were used to examine associations of changes in PDI, hPDI, and uPDI with changes in log-transformed biomarker concentrations between the 2 time points. For each quintile of index changes, least squares geometric mean percentage changes in biomarkers were calculated and adjusted for baseline age, baseline BMI (in quintiles), baseline corresponding PDI (in quintiles), baseline corresponding biomarker concentrations (in quintiles), as well as changes in total energy intake (in quintiles), alcohol intake (in quintiles), smoking status, physical activity (in quintiles), menopausal status, postmenopausal hormone use, hypertension, and hypercholesterolemia. We further adjusted for weight change (in quintiles) in a separate model. Linear trends were tested using the median of each index change within each quintile as a continuous variable. The results were expressed as percentage differences of the percentage changes in biomarker concentrations per 10-point difference in diet index changes, and visualized in a bar graph. Additional analyses evaluated whether the associations were modified by baseline BMI (<25, 25–29.9, ≥30.0) or weight change (less than the median of 2.7 kg or ≥2.7 kg). The significance of interactions was evaluated by testing the cross product term between changes in the three PDIs and baseline BMI or weight change. Further, we provided information on changes in individual food group intakes according to the quintiles of changes in PDI scores, and the trends were evaluated based on univariate general linear regression models using each index score change as a continuous variable.

Analyses were performed in SAS 9.4 (SAS Institute Inc.), and *P* values <0.05 were considered significant.

## Results

Age-standardized characteristics of participants at baseline and follow-up are shown in **Table 1**. At baseline, participants were, on average, 45 y old and their mean BMI was 24.6. During an average follow-up of 13 y (range: 11–15 y), mean plasma concentrations of all biomarkers increased. In addition, participants on average had increased BMI (24.6 to 26.3), alcohol intake (3.9 to 6.2 g/day), physical activity (17.8 to 23.5 MET-h/wk), postmenopausal status (20.6% to 72.1%), prevalence of hypertension (10.9% to 29.4%) and hypercholesterolemia (15.1% to 35.1%), decreased total energy intake (1886 to 1756 kcal/d), current smoking (4.1% to 1.5%), and postmenopausal hormone use (85.5% to 23.9%). About 59.8% of women had an increased PDI score during the follow-up, compared with 46.0% for an increased hPDI score and 15.4% for an increased uPDI score.

**FIGURE 1:**
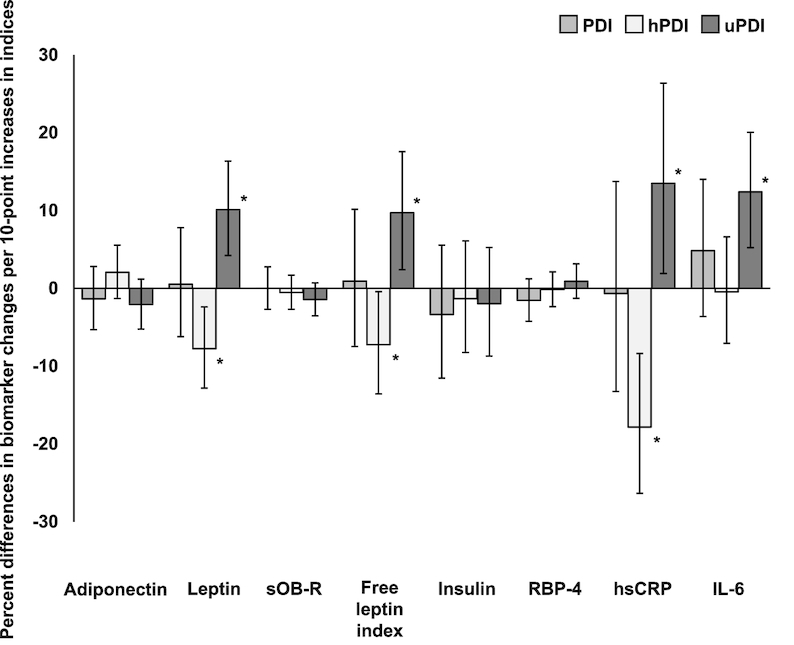
Percent differences and 95% CIs of plasma adiposity-associated biomarker concentration changes per 10-point increase of plant-based diet indices in the Nurses’ Health Study II (*n* = 831). Error bars indicate 95% CIs, and asterisks indicate *P* < 0.05 for the associations between biomarker changes and changes in plant-based diet indices (per 10-point), after adjustment for baseline age, baseline corresponding PDIs, baseline corresponding biomarker concentrations, baseline BMI, and changes in weight, total energy intake, alcohol intake, smoking status, physical activity, menopausal status, postmenopausal hormone use, hypertension, and hypercholesterolemia. hPDI, healthful plant-based diet index; hsCRP, high-sensitivity C-reactive protein; PDI, overall plant-based diet index; RBP-4, retinol-binding protein-4; sOB-R, soluble leptin receptor; uPDI, unhealthful plant-based index.

**TABLE 1:** Comparison of age-standardized characteristics between baseline (1996–1999) and follow-up (2010–2011) in the Nurses’ Health Study II^1^

	Baseline	Follow-up	*P*
*n*	831	831	
Age at blood collection,^2^ y	45 ± 5	58 ± 4	<0.001
BMI, kg/m^2^	24.6 ± 3.5	26.3 ± 4.7	<0.001
Weight, kg	66.4 ± 10.2	70.7 ± 12.8	<0.001
Total energy intake, kcal/day	1886 ± 364	1756 ± 409	<0.001
Alcohol, g/day	3.9 ± 5.8	6.2 ± 8.2	<0.001
Current smokers, %	4.1	1.5	<0.001
Physical activity, MET-h/wk	17.8 ± 15.2	23.5 ± 22.2	<0.001
Postmenopausal, %	20.6	72.1	<0.001
Current PMH use in postmenopausal, %	85.5	23.9	<0.001
Hypertension, %	10.9	29.4	<0.001
Hypercholesterolemia, %	15.1	35.1	<0.001
PDI	55.9 ± 4.8	57.8 ± 4.7	<0.001
hPDI	55.2 ± 5.7	54.3 ± 6.2	0.60
uPDI	54.0 ± 5.7	48.0 ± 5.8	<0.001
Plasma biomarkers			
Total adiponectin, µg/mL	7.0 [1.4]	7.4 [1.5]	<0.001
Leptin, ng/mL	17.0 [1.8]	19.4 [2.0]	<0.001
Soluble leptin receptor, ng/mL	24.8 [1.2]	25.0 [1.3]	<0.001
Free leptin index^3^	0.7 [2.0]	0.8 [2.3]	0.001
Insulin, µU/mL	5.1 [1.6]	5.5 [1.7]	0.11
RBP-4, µg/mL	32.8 [1.2]	35.7 [1.2]	<0.001
High-sensitivity CRP, mg/L	0.99 [2.55]	1.03 [2.58]	0.10
IL-6, pg/mL	0.87 [1.54]	0.91 [1.57]	0.02

^1^Values are means ± SDs for continuous variables except plasma biomarkers, geometric means [geometric SDs] for all plasma biomarkers, and percentages for categorical variables; and are standardized to the age distribution of participants. *P* value is calculated based on paired *t* test (for continuous variables) or McNemar's test (for binary variables). CRP, C-reactive protein; hPDI, healthful plant-based diet index; MET-h, metabolic equivalent task hours; PDI, overall plant-based diet index; PMH, postmenopausal hormone; RBP-4, retinol-binding protein-4; uPDI, unhealthful plant-based diet index.

^2^Value is not age adjusted.

^3^Free leptin index is defined as the ratio of leptin to soluble leptin receptor.

Changes in food group intakes by quintiles of changes in PDI, hPDI, and uPDI are shown in **Supplemental Table 1**. Increased PDIs and hPDI were mainly attributed to increased intake of healthy plant foods, whereas increased uPDI was related to decreased intake of healthy plant foods. In general, intake of less healthy plant foods decreased over follow-up, with more remarkable reductions observed for women who had increased hPDI than women who had increased PDI or uPDI. Increased PDI, hPDI, and uPDI were associated with decreased intake of animal foods.

In cross-sectional analyses, higher hPDI was significantly associated with lower leptin (*P*-trend < 0.001), free leptin index (*P*-trend < 0.001), insulin (*P*-trend < 0.001), hsCRP (*P*-trend < 0.001), and higher adiponectin (*P*-trend = 0.025) and sOB-R (*P*-trend = 0.024) after adjusting for all covariates including BMI (**Table 2**). The multivariable-adjusted percentage changes in biomarker concentrations (95% CI) for every 10-point higher hPDI were −7.2% (−11.0, −3.1) for leptin, −8.8% (−13.3, −3.9) for free leptin index, −10.0% (−14.2, −5.6) for insulin, −13.6% (−20.5, −6.1) for hsCRP, 3.0% (0.4, 5.7) for adiponectin, and 1.9% (0.3, 3.7) for sOB-R. By contrast, higher uPDI was significantly associated with a higher concentration of leptin (percentage change for every 10-point higher uPDI: 4.4%, 95% CI: 0.2, 8.6; *P*-trend = 0.037) and insulin (percentage change for every 10-point higher uPDI: 4.8%, 95% CI: 0.1, 9.7; *P*-trend = 0.048, Table 2). Higher uPDI was significantly associated with a higher free leptin index (*P*-trend = 0.002) after adjusting for potential covariates without BMI. However, this association was attenuated after adjustment for BMI (*P*-trend = 0.14). Further, a higher PDI was significantly associated with lower concentrations of insulin (*P*-trend = 0.007) and hsCRP (*P*-trend = 0.018) after adjusting for covariates without BMI (**Supplemental Table 2**), but these associations were also attenuated after adjustment for BMI (*P*-trend = 0.15 for insulin and 0.12 for hsCRP).

**TABLE 2:** Cross-sectional associations of plasma adiposity-associated biomarker concentrations with quintiles of an hPDI and a uPDI in 1995 and 2011 in the Nurses’ Health Study II^1^

	Quintile 1	Quintile 2	Quintile 3	Quintile 4	Quintile 5	Percentage difference/10-point higher indices (95% CIs)	*P*-trend
hPDI							
Baseline, *n*	165	159	205	146	156		
Follow-up, *n*	172	137	202	158	162		
Median (range)	46 (32–48)	51 (49–52)	55 (53–57)	59 (58–61)	65 (62–81)		
Adiponectin, ng/mL							
Multivariable^2^	6.9 (6.5, 7.3)	7.2 (6.8, 7.6)	7.2 (6.8, 7.6)	7.4 (7.0, 7.9)	7.4 (7.0, 7.9)	3.9 (1.2, 6.7)	0.005
Multivariable + BMI	6.8 (6.4, 7.2)	7.1 (6.7, 7.5)	7.1 (6.7, 7.5)	7.3 (6.9, 7.7)	7.2 (6.8, 7.7)	3.0 (0.4, 5.7)	0.025
Leptin, ng/mL							
Multivariable	20.0 (17.9, 22.3)	18.3 (16.5, 20.4)	18.4 (16.6, 20.4)	17.2 (15.4, 19.1)	15.9 (14.2, 17.7)	−10.6 (−15.1, −6.0)	<0.001
Multivariable + BMI	19.3 (17.7, 21.1)	17.7 (16.2, 19.2)	17.9 (16.5, 19.5)	17.6 (16.2, 19.2)	16.4 (15.0, 17.9)	−7.2 (−11.0, −3.1)	<0.001
sOB-R, ng/mL							
Multivariable	23.4 (22.5, 24.3)	24.3 (23.4, 25.2)	24.3 (23.4, 25.1)	25.2 (24.3, 26.1)	24.8 (23.9, 25.7)	2.8 (1.0, 4.6)	0.002
Multivariable + BMI	23.4 (22.6, 24.2)	24.2 (23.3, 25.0)	24.0 (23.3, 24.8)	24.7 (23.8, 25.5)	24.3 (23.4, 25.1)	1.9 (0.3, 3.7)	0.024
Free leptin index^3^							
Multivariable	0.8 (0.7, 1.0)	0.8 (0.7, 0.9)	0.8 (0.7, 0.8)	0.7 (0.6, 0.8)	0.6 (0.6, 0.7)	−12.8 (−18.0, −7.3)	<0.001
Multivariable + BMI	0.8 (0.7, 0.9)	0.7 (0.7, 0.8)	0.7 (0.7, 0.8)	0.7 (0.6, 0.8)	0.7 (0.6, 0.8)	−8.8 (−13.3, −3.9)	<0.001
Insulin, µU/mL							
Multivariable	6.3 (5.7, 7.0)	5.6 (5.1, 6.2)	5.5 (5.0, 6.1)	5.0 (4.5, 5.6)	4.8 (4.3, 5.3)	−12.7 (−17.1, −8.1)	<0.001
Multivariable + BMI	6.2 (5.7, 6.9)	5.7 (5.2, 6.2)	5.5 (5.1, 6.1)	5.3 (4.8, 5.8)	5.1 (4.6, 5.6)	−10.0 (−14.2, −5.6)	<0.001
RBP-4, µg/mL							
Multivariable	36.5 (35.4, 37.7)	36.0 (34.9, 37.2)	36.3 (35.3, 37.5)	36.0 (34.9, 37.2)	36.4 (35.2, 37.6)	−0.4 (−2.0, 1.2)	0.61
Multivariable + BMI	36.4 (35.2, 37.6)	35.8 (34.7, 37.0)	36.2 (35.1, 37.3)	35.9 (34.8, 37.1)	36.2 (35.0, 37.4)	−0.1 (−1.7, 1.5)	0.90
hsCRP, mg/L							
Multivariable	1.21 (1.00, 1.45)	1.15 (0.96, 1.38)	1.03 (0.87, 1.23)	0.85 (0.71, 1.02)	0.87 (0.72, 1.05)	−18.1 (−25.1, −10.5)	<0.001
Multivariable + BMI	1.17 (0.98, 1.39)	1.15 (0.97, 1.37)	1.04 (0.88, 1.22)	0.91 (0.77, 1.07)	0.93 (0.78, 1.10)	−13.6 (−20.5, −6.1)	<0.001
IL-6, pg/mL							
Multivariable	0.93 (0.84, 1.02)	0.89 (0.81, 0.97)	0.87 (0.80, 0.95)	0.89 (0.81, 0.97)	0.87 (0.79, 0.96)	−2.9 (−7.4, 1.8)	0.22
Multivariable + BMI	0.91 (0.83, 0.99)	0.88 (0.81, 0.97)	0.87 (0.80, 0.94)	0.90 (0.83, 0.99)	0.88 (0.81, 0.97)	−0.7 (−5.1, 3.9)	0.75
uPDI							
Baseline, *n*	64	118	156	207	286		
Follow-up, *n*	255	224	156	123	73		
Median (range)	40 (28–43)	46 (44–48)	50 (49–52)	55 (53–57)	61 (58–77)		
Adiponectin, ng/mL							
Multivariable	7.4 (7.0, 7.9)	7.4 (7.0, 7.8)	7.0 (6.6, 7.5)	7.0 (6.7, 7.5)	7.1 (6.7, 7.6)	−2.4 (−4.8, 0.1)	0.06
Multivariable + BMI	7.2 (6.8, 7.7)	7.3 (6.9, 7.7)	6.9 (6.6, 7.3)	7.0 (6.6, 7.4)	7.0 (6.6, 7.5)	−1.6 (−4.0, 0.8)	0.19
Leptin, ng/mL							
Multivariable	16.0 (14.4, 17.9)	17.6 (15.9, 19.6)	18.2 (16.4, 20.2)	19.2 (17.3, 21.4)	19.3 (17.3, 21.6)	9.2 (4.1, 14.5)	<0.001
Multivariable + BMI	17.0 (15.5, 18.5)	17.5 (16.1, 19.0)	18.1 (16.6, 19.6)	18.2 (16.7, 19.8)	18.6 (17.0, 20.3)	4.4 (0.2, 8.6)	0.037
sOB-R, ng/mL							
Multivariable	24.8 (23.9, 25.7)	24.4 (23.5, 25.3)	24.0 (23.1, 24.9)	24.1 (23.2, 25.0)	24.0 (23.1, 25.0)	−1.3 (−3.0, 0.3)	0.11
Multivariable + BMI	24.3 (23.5, 25.2)	24.2 (23.4, 25.1)	23.8 (23.0, 24.6)	24.1 (23.3, 24.9)	24.0 (23.2, 24.9)	−0.6 (−2.1, 1.0)	0.48
Free leptin index							
Multivariable	0.6 (0.6, 0.7)	0.7 (0.6, 0.8)	0.8 (0.7, 0.9)	0.8 (0.7, 0.9)	0.8 (0.7, 0.9)	9.8 (3.7, 16.3)	0.002
Multivariable + BMI	0.7 (0.6, 0.8)	0.7 (0.7, 0.8)	0.8 (0.7, 0.8)	0.7 (0.7, 0.8)	0.8 (0.7, 0.8)	3.7 (−1.2, 8.9)	0.14
Insulin, µU/mL							
Multivariable	5.0 (4.5, 5.6)	5.6 (5.0, 6.2)	5.6 (5.1, 6.2)	5.6 (5.1, 6.2)	5.7 (5.1, 6.3)	5.3 (0.2, 10.7)	0.043
Multivariable + BMI	5.1 (4.6, 5.6)	5.7 (5.2, 6.2)	5.7 (5.2, 6.3)	5.7 (5.2, 6.2)	5.7 (5.2, 6.3)	4.8 (0.1, 9.7)	0.048
RBP-4, µg/mL							
ultivariable	35.5 (34.4, 36.7)	36.2 (35.1, 37.3)	35.8 (34.7, 36.9)	36.6 (35.5, 37.8)	36.6 (35.4, 37.8)	1.4 (−0.1, 3.0)	0.06
Multivariable + BMI	35.5 (34.4, 36.7)	36.2 (35.0, 37.3)	35.8 (34.7, 36.9)	36.5 (35.4, 37.7)	36.5 (35.4, 37.7)	1.3 (−0.2, 2.8)	0.09
hsCRP, mg/L							
Multivariable	0.90 (0.75, 1.09)	1.00 (0.84, 1.20)	1.10 (0.92, 1.31)	1.10 (0.91, 1.31)	1.03 (0.85, 1.24)	6.7 (−2.0, 16.1)	0.14
Multivariable + BMI	0.96 (0.80, 1.14)	1.03 (0.87, 1.21)	1.12 (0.95, 1.33)	1.08 (0.91, 1.28)	1.02 (0.85, 1.21)	3.3 (−4.6, 11.8)	0.42
IL-6, pg/mL							
Multivariable	0.86 (0.79, 0.95)	0.89 (0.82, 0.98)	0.89 (0.82, 0.98)	0.90 (0.82, 0.99)	0.90 (0.82, 0.99)	1.6 (−2.9, 6.3)	0.50
Multivariable + BMI	0.87 (0.79, 0.95)	0.89 (0.82, 0.98)	0.90 (0.82, 0.98)	0.90 (0.82, 0.98)	0.89 (0.81, 0.98)	1.1 (−3.1, 5.6)	0.61

^1^Values are least squares geometric means (95% CIs) from linear mixed model to account for within-person correlation between the 2 blood-measurement time points. hPDI, healthful plant-based diet index; hsCRP, high-sensitivity C-reactive protein; RBP-4, retinol-binding protein-4; sOB-R, soluble leptin receptor; uPDI, unhealthful plant-based diet index.

^2^Adjusted for age, time period, total energy intake, alcohol intake, smoking status, physical activity, menopausal status, postmenopausal hormone use, hypertension, and hypercholesterolemia.

^3^Free leptin index is defined as the ratio of leptin to soluble leptin receptor.

The associations between changes in hPDI and uPDI and percentage changes in biomarker concentrations are shown in **Table 3** and **Figure 1**. hPDI increase was significantly associated with smaller increases in leptin (*P*-trend = 0.005) and free leptin index (*P*-trend = 0.038), and a larger decrease in hsCRP (*P*-trend < 0.001) after adjusting for all covariates including weight change (Table 3). The multivariable-adjusted percentage differences in biomarker changes (95% CI) for every 10-point hPDI increase were −7.7% (−12.8, −2.4) for leptin, −7.2% (−13.6, −0.4) for free leptin index, and −17.8% (−26.3, −8.4) for hsCRP (Figure 1). Similarly, hPDI increase was positively associated with a larger increase in adiponectin prior to adjustment for weight change (*P*-trend = 0.020), but the association was attenuated after the adjustment (*P*-trend = 0.23). In contrast, uPDI increase was significantly associated with larger increases in leptin (*P*-trend < 0.001), free leptin index (*P*-trend = 0.009), hsCRP (*P*-trend = 0.021), and IL-6 (*P*-trend < 0.001) after adjusting for all covariates including weight change (Table 3). The multivariable-adjusted percentage differences in biomarker changes (95% CI) for every 10-point uPDI increase were 10.1% (4.2, 16.4) for leptin, 9.7% (2.4, 17.6) for free leptin index, 13.5% (1.9, 26.4) for hsCRP, and 12.4% (5.2, 20.1) for IL-6 (Figure 1). Associations of uPDI increase with decreases in adiponectin (*P*-trend = 0.025) and sOB-R (*P*-trend = 0.018) were attenuated after adjusting for weight change. There were no significant associations between PDI change and percentage changes in biomarkers (**Supplemental Table 3**). In general, these associations did not differ substantially by baseline BMI and weight change (data not shown).

**TABLE 3:** Percentage changes in plasma adiposity-associated biomarker concentrations across quintiles of an hPDI change and a uPDI change between 1995 and 2011 in the Nurses’ Health Study II^1^

	Quintile 1	Quintile 2	Quintile 3	Quintile 4	Quintile 5	*P*-trend
hPDI						
* n*	174	174	146	181	156	
Median (range)	−9 (−20, −6)	−3 (−5, −2)	0 (−1, 1)	4 (2, 6)	10 (7, 24)	
Adiponectin						
Multivariable^2^	−2.3 (−10.1, 6.1)	3.9 (−4.4, 12.9)	−0.3 (−8.5, 8.5)	2.6 (−5.5, 11.4)	7.4 (−1.5, 17.1)	0.020
Multivariable + Wt change	1.8 (−6.0, 10.2)	6.6 (−1.5, 15.4)	3.2 (−4.8, 12.0)	3.0 (−4.8, 11.5)	7.9 (−0.6, 17.1)	0.23
Leptin						
Multivariable	58.1 (34.9, 85.2)	41.4 (20.7, 65.7)	32.6 (12.7, 56.0)	27.5 (8.9, 49.2)	16.8 (−0.9, 37.6)	<0.001
Multivariable + Wt change	37.4 (20.2, 57.1)	29.8 (13.6, 48.3)	18.6 (3.4, 36.0)	26.3 (10.7, 44.2)	16.3 (1.3, 33.5)	0.005
sOB-R						
Multivariable	−4.5 (−9.5, 0.9)	−2.2 (−7.4, 3.3)	−3.6 (−8.9, 1.9)	−1.9 (−7.1, 3.5)	−2.8 (−8.1, 2.9)	0.44
Multivariable + Wt change	−2.0 (−7.0, 3.2)	−0.6 (−5.6, 4.7)	−1.4 (−6.6, 4.0)	−1.8 (−6.7, 3.5)	−2.6 (−7.8, 2.8)	0.65
Free leptin index^3^						
Multivariable	66.0 (36.8, 101.6)	45.4 (19.8, 76.4)	33.9 (9.7, 63.4)	31.2 (8.2, 59.1)	21.0 (−1.0, 47.9)	<0.001
Multivariable + Wt change	42.1 (20.2, 68.1)	33.0 (12.5, 57.1)	18.5 (−0.3, 40.7)	31.2 (11.1, 54.8)	21.0 (1.8, 43.9)	0.038
Insulin						
Multivariable	30.5 (8.8, 56.6)	24.3 (3.8, 49.0)	31.8 (9.5, 58.7)	18.1 (−1.3, 41.3)	17.3 (−2.7, 41.4)	0.12
Multivariable + Wt change	20.7 (1.4, 43.8)	18.8 (−0.1, 41.2)	23.9 (3.7, 48.0)	18.9 (0.2, 41.2)	17.4 (−1.8, 40.3)	0.72
RBP-4						
Multivariable	13.1 (7.2, 19.3)	10.5 (4.8, 16.5)	10.0 (4.1, 16.2)	12.6 (6.8, 18.8)	10.8 (4.8, 17.1)	0.62
Multivariable + Wt change	12.2 (6.4, 18.4)	10.0 (4.3, 16.0)	9.3 (3.5, 15.5)	12.6 (6.8, 18.8)	10.8 (4.9, 17.1)	0.91
hsCRP						
Multivariable	35.0 (3.1, 76.7)	3.5 (−20.9, 35.3)	1.4 (−23.0, 33.6)	−2.6 (−25.4, 27.2)	−22.4 (−41.3, 2.5)	<0.001
Multivariable + Wt change	20.2 (−7.3, 55.7)	−3.9 (−25.7, 24.3)	−7.6 (−29.1, 20.5)	−2.8 (−24.7, 25.5)	−22.2 (−40.4, 1.6)	<0.001
IL-6						
Multivariable	14.7 (−2.4, 34.8)	13.2 (−3.7, 33.0)	21.1 (2.6, 42.9)	8.8 (−7.4, 27.8)	12.5 (−4.9, 32.9)	0.61
Multivariable + Wt change	12.2 (−4.5, 31.9)	11.8 (−4.8, 31.4)	18.5 (0.4, 40.0)	8.7 (−7.4, 27.6)	12.6 (−4.7, 33.0)	0.90
uPDI						
*n*	157	174	148	192	160	
Median (range)	−17 (−31, −14)	−11 (−13, −9)	−7 (−8, −6)	−3 (−5, −1)	3 (0, 20)	
Adiponectin						
Multivariable	6.6 (−2.2, 16.2)	3.2 (−5.0, 12.2)	0.6 (−7.4, 9.4)	0.0 (−8.0, 8.6)	−1.5 (−9.6, 7.2)	0.025
Multivariable + Wt change	7.6 (−0.8, 16.8)	4.0 (−4.0, 12.6)	2.6 (−5.2, 11.1)	3.7 (−4.1, 12.3)	2.3 (−5.7, 11.0)	0.21
Leptin						
Multivariable	14.7 (−2.7, 35.1)	22.2 (4.3, 43.2)	45.5 (24.1, 70.5)	42.6 (21.8, 66.8)	57.2 (33.6, 84.9)	<0.001
Multivariable + Wt change	12.2 (−2.2, 28.7)	19.7 (4.8, 36.8)	36.2 (19.3, 55.6)	27.3 (11.5, 45.3)	38.4 (20.7, 58.7)	<0.001
sOB-R						
Multivariable	0.7 (−4.8, 6.5)	−1.7 (−6.9, 3.8)	−3.8 (−8.9, 1.6)	−4.1 (−9.2, 1.2)	−4.6 (−9.8, 0.8)	0.018
Multivariable + Wt change	1.0 (−4.2, 6.6)	−1.4 (−6.4, 3.9)	−2.7 (−7.6, 2.5)	−1.9 (−6.9, 3.3)	−2.3 (−7.4, 3.1)	0.19
Free leptin index						
Multivariable	14.7 (−6.2, 40.1)	26.7 (4.3, 53.9)	53.9 (26.7, 86.9)	49.3 (23.2, 81.1)	59.7 (30.9, 94.8)	<0.001
Multivariable + Wt change	12.5 (−5.3, 33.6)	25.0 (5.7, 47.7)	44.4 (22.1, 70.7)	31.9 (11.7, 55.8)	38.6 (16.7, 64.6)	0.009
Insulin						
Multivariable	26.8 (5.1, 52.9)	19.5 (−0.5, 43.6)	18.9 (−0.8, 42.5)	20.8 (0.9, 44.8)	32.7 (10.1, 59.9)	0.53
Multivariable + Wt change	26.4 (5.8, 51.0)	19.2 (0.1, 41.9)	15.3 (−3.0, 37.0)	14.2 (−3.9, 35.7)	22.5 (2.4, 46.4)	0.59
RBP-4						
Multivariable	9.1 (3.3, 15.3)	11.4 (5.7, 17.5)	12.4 (6.5, 18.6)	12.7 (6.8, 18.8)	12.0 (6.1, 18.4)	0.25
Multivariable + Wt change	9.0 (3.2, 15.2)	11.3 (5.5, 17.4)	12.0 (6.2, 18.1)	12.0 (6.1, 18.1)	11.2 (5.3, 17.5)	0.41
hsCRP						
Multivariable	−17.0 (−37.3, 9.7)	−4.9 (−27.5, 24.7)	15.6 (−11.9, 51.6)	5.6 (−19.4, 38.1)	24.6 (−5.6, 64.6)	0.001
Multivariable + Wt change	−18.0 (−37.3, 7.1)	−6.0 (−27.4, 21.9)	9.9 (−15.3, 42.4)	−4.3 (−26.1, 23.9)	11.5 (−14.7, 45.6)	0.021
IL-6						
Multivariable	1.6 (−13.8, 19.7)	7.0 (−8.9, 25.6)	13.5 (−3.3, 33.1)	19.4 (1.9, 40.0)	29.8 (10.2, 52.9)	<0.001
Multivariable + Wt change	1.3 (−14.0, 19.4)	7.0 (−8.8, 25.6)	12.6 (−4.0, 32.1)	17.2 (0.0, 37.5)	28.1 (8.7, 51.1)	<0.001

^1^Values are adjusted least squares percentage changes (95% CIs) in plasma biomarker concentrations from general linear model. hPDI, healthful plant-based diet index; hsCRP, high-sensitivity C-reactive protein; RBP-4, retinol-binding protein-4; sOB-R, soluble leptin receptor; uPDI, unhealthful plant-based diet index; Wt, weight.

^2^Adjusted for age at the first blood measurement, changes in total energy intake, alcohol intake, smoking status, physical activity, menopausal status, postmenopausal hormone use, hypertension, hypercholesterolemia, baseline BMI, baseline corresponding plant-based diet indices, and baseline corresponding biomarker concentrations.

^3^Free leptin index is defined as the ratio of leptin to soluble leptin receptor.

## Discussion

In the cross-sectional analyses, we found that a higher hPDI score—a measure of adherence to a high-quality plant-based diet—was significantly associated with lower plasma concentrations of leptin, insulin, and hsCRP, and a lower free leptin index, and higher plasma concentrations of adiponectin and sOB-R, even after adjustment for BMI and other covariates. However, a higher uPDI score—a measure of adherence to a low-quality plant-based diet—was significantly associated with higher concentrations of leptin and insulin. In the longitudinal analyses adjusted for weight change and other covariates, women who improved their adherence to the healthful plant-based diet over 13 y of follow-up, as measured by increased hPDI scores, had smaller increases in leptin concentration and free leptin index, and a larger decrease in hsCRP concentration. In addition, women with increased uPDI scores had significant increases in leptin concentration, hsCRP concentration, IL-6 concentration, and free leptin index. The consistency between cross-sectional and longitudinal evidence strongly supports the potential benefits of a healthful plant-based diet on metabolic and inflammatory profiles.

Our results provide supporting evidence for the anti-inflammatory mechanisms through which a healthy plant-based dietary pattern is associated with lower risk of CHD and T2D (21, 22). In this study we observed consistently lower leptin concentrations with higher hPDI and lower uPDI cross-sectionally, and with increase in hPDI and decrease in uPDI longitudinally. Leptin is well known for not only inhibiting appetite by transmitting signals to hypothalamic cells (27), but also acting as a proinflammatory cytokine and stimulating the production of inflammatory mediators such as IL-1, IL-6, IL-12, and TNF (6). In the cross-sectional analysis, we also observed that sOB-R, which binds to leptin and regulates bioavailability of free leptin, had a significant positive association with hPDI, although this association was not observed in the longitudinal analysis. Similarly, plasma hsCRP concentrations were inversely associated with higher hPDI cross-sectionally, and with increase in hPDI and decrease in uPDI longitudinally, indicating lower inflammatory profiles among women who adhered to or improved the healthy plant-based diet. Further, our results suggest a possible inverse relationship between a healthy plant-based diet and leptin resistance, a metabolic phenotype in obese individuals characterized by substantially elevated leptin concentration due to the blocked leptin activity (28). Recent research on leptin resistance has revealed that CRP can competitively bind to sOB-R and directly inhibit the binding of leptin to sOB-R (29, 30). Interestingly, IL-6 showed a significant positive association only with uPDI change, but not with hPDI or hPDI change. It is possible that adherence to an unhealthy plant-based diet could have a particularly strong detrimental effect on IL-6, a marker of inflammation and insulin resistance (31).

The anti-inflammatory and anti–insulin resistance properties have been reported for the nutrients particularly associated with healthy plant foods. For example, in experimental models, polyphenols, such as procyanidin and cyanidin 3-glucoside found in fruits and vegetables, act as antioxidants inhibiting the production of proinflammatory cytokines, including CRP and IL-6 (32, 33). Anthocyanin and flavonol intakes have also been associated with a lower inflammation score that is calculated by 12 inflammatory biomarkers in US adults (34). In addition, dietary fiber intake has been inversely associated with hsCRP (35) and IL-6 (36). Conversely, *trans* fat, commonly found in less healthy plant foods, has been associated with elevated oxidative stress and inflammation (37). Saturated fat can increase the expression of inflammatory cytokines, including IL-6 (38). A diet rich in saturated fat also leads to a predominantly gram-negative lipopolysaccharide-rich gut microbial pattern, which promotes inflammation (39). Particularly, the specific positive correlation of IL-6 with plasma endotoxin, which is modulated by dietary saturated fat (39), might help to explain why IL-6 was only associated with uPDI in this study.

Our results were consistent with several previous studies that examined the associations between dietary patterns and biomarkers. The Mediterranean diet is characterized by a high intake of vegetables, legumes, fruits, nuts, refined cereals, and olive oil; a low intake of saturated lipids, meat, and poultry; a moderately high intake of fish; a low-to-moderate intake of dairy products; and a regular but moderate intake of wine (40). After a 2-y follow-up, the Mediterranean diet was associated with decreased hsCRP, IL-6, and insulin resistance in patients with metabolic syndrome (41); and associated with increased adiponectin and decreased CRP in T2D patients (42). The Dietary Approaches to Stop Hypertension (DASH)-style diet is a dietary pattern that recommends high intake of fruits, vegetables, whole grains, poultry, fish, and nuts, and restricts saturated fat, red meat, sweet beverages, and refined grains (43). A meta-analysis with 451 participants showed that the DASH-style diet reduced serum hsCRP concentrations (44). To our knowledge, the only study examining the association between diet and sOB-R or free leptin index was a previous publication investigating the association between the Alternate Health Eating Index-2010 (AHEI-2010) and biomarkers (23). The AHEI-2010 is a dietary score that has higher scores assigned to higher intakes of vegetables, fruit, whole grains, nuts/legumes, long-chain fats, and PUFAs, and moderate alcohol consumption; and lower intakes of sugar-sweetened beverages, red/processed meat, *trans* fat, and sodium (23). Both the current study and the previous publication on AHEI-2010 showed that a healthy diet was positively associated with sOB-R and inversely associated with free leptin index. In addition, we found similar associations between healthy diet and adiponectin, leptin, and insulin. Moreover, only in our study was there a significant association between healthy diet and hsCRP, even after adjusting for BMI or weight change. Because of the emerging evidence of associations with obesity-related diseases and inflammation (45–47), when compared with the AHEI-2010, the hPDI additionally accounts for lowered intake of less healthy plant-based food, such as refined grains, potatoes, and sweets, possibly contributing to a stronger association between hPDI and hsCRP. In this study, the PDI had no significant associations with adiposity-associated biomarkers, either cross-sectionally or longitudinally. The result is consistent with previous publications showing that a hPDI has a stronger association with a reduced risk of developing CHD and T2D (21, 22), and emphasizes the importance of differentiating the quality of plant-based food. The opposite associations we observed between hPDI and uPDI for multiple inflammatory and metabolic biomarkers corroborate the recommendation that increasing the intake of healthy plant foods and reducing the intake of less healthy plant foods simultaneously would be essential for the prevention of cardiometabolic diseases.

Several limitations should be noted. First, the participants were predominantly Caucasian registered nurses without CVD and T2D. Therefore, translating results to the general population should be done with caution. Second, although we have controlled for multiple important lifestyle factors in the analyses, the possibility of residual confounding cannot be excluded. Third, our dietary assessment was based on self-reported questionnaires, which could introduce measurement errors. However, the consistency of the strong associations between diet indices and biomarkers in both cross-sectional and longitudinal analyses suggests that the observed associations were unlikely to be entirely explained by residual confounding or measurement errors. Further, the potential impact of long-term blood storage on the biomarker stability needs additional studies, which might reveal nondifferential measurement errors and likely attenuate the observed associations.

In conclusion, adherence to a healthful plant-based diet is associated with favorable changes in adiposity-related inflammatory and metabolic biomarkers even after adjustment for weight change. Our results provide evidence for biological mechanisms underlying the inverse associations between a healthful plant-based diet and obesity-related diseases, and support current recommendations to increase intake of healthy plant foods, while reducing intake of less healthy plant foods for improved health outcomes.

## Supplementary Material

nxy301_Supplement_Tables

## Notes

Supported by NIH grants UM1 CA176726, R01 CA67262, HL60712, and P30 DK46200. Dr Baden is supported by Manpei Suzuki Diabetes Foundation. Dr Satija is supported by American Heart Association Grant #16POST29660000.

Author disclosures: FBH, reported receiving research support from California Walnut Commission and lecture fees from Standard Process. MYB, AS, and TH, no conflicts of interest.

Supplemental Tables 1–3 are available from the “Supplementary data” link in the online posting of the article and from the same link in the online table of contents at https://academic.oup.com/jn/.

Abbreviations used: AHEI-2010, Alternate Health Eating Index-2010; CHD, coronary heart disease; CRP, C-reactive protein; FFQ, food frequency questionnaire; hPDI, healthful plant-based diet index; hsCRP, high-sensitivity C-reactive protein; IL-6, interleukin-6; PDI, overall plant-based diet index; RBP-4, retinol-binding protein-4; sOB-R, soluble leptin receptor; T2D, type 2 diabetes; uPDI, unhealthful plant-based diet index.
