# Supplementary material for: Change in Plant-Based Diet Quality Is Associated with Changes in Plasma Adiposity-Associated Biomarker Concentrations in Women
Source: J Nutr. 2019 Mar 30;149(4):676–86. doi: 10.1093/jn/nxy301 (PMC6461739; doi:10.1093/jn/nxy301)
Supplement: nxy301_Supplement_Tables [file nxy301_supplement_tables.pdf]

Supplementary data

Supplemental Table 1 Changes in food group intakes among quintiles of changes in plant-based diet indices in the Nurses' Health study II[1]

	Quintile 1	Quintile 2	Quintile 3	Quintile 4	Quintile 5	*P*
**Overall plant-based diet index (PDI)**						
n	161	162	197	131	180	
Median (range)	-6 (-18, -4)	-1.5 (-3, 0)	3 (1, 4)	6 (5, 7)	10 (8, 27)	
Healthy plant foods, servings/day						
Whole grains	-0.7 ± 1.4	-0.2 ± 1.2	-0.1 ± 1.4	0.0 ± 1.1	0.2 ± 1.2	<0.001
Fruits	0.0 ± 1.2	0.3 ± 1.2	0.4 ± 1.1	0.3 ± 0.9	0.5 ± 0.9	<0.001
Vegetables	-0.3 ± 2.1	0.4 ± 1.8	0.5 ± 2.2	0.6 ± 1.9	0.9 ± 1.7	<0.001
Nuts	0.5 ± 0.9	0.6 ± 1.0	0.7 ± 1.2	0.5 ± 0.8	0.7 ± 1.0	0.08
Legumes	-0.1 ± 0.6	0.0 ± 0.5	0.1 ± 0.5	0.2 ± 0.7	0.2 ± 0.4	<0.001
Vegetable oil	0.3 ± 0.6	0.4 ± 0.6	0.3 ± 0.7	0.4 ± 0.7	0.5 ± 0.6	0.007
Tea and coffee	-0.2 ± 1.9	-0.3 ± 1.8	0.1 ± 1.8	0.1 ± 1.6	0.3 ± 1.7	0.001
Less healthy plant foods, servings/day						
Fruit juices	-0.6 ± 0.8	-0.4 ± 0.6	-0.3 ± 0.6	-0.2 ± 0.6	-0.1 ± 0.9	<0.001
Refined grains	-1.1 ± 1.3	-1.1 ± 1.3	-0.8 ± 1.3	-0.7 ± 1.2	-0.5 ± 1.1	<0.001
Potatoes	-0.2 ± 0.4	-0.2 ± 0.3	-0.1 ± 0.3	-0.1 ± 0.3	0.0 ± 0.3	<0.001
Sugar-sweetened beverages	-0.4 ± 1.0	-0.2 ± 0.6	-0.2 ± 0.6	-0.2 ± 0.7	-0.1 ± 0.7	<0.001
Sweets and desserts	-0.8 ± 1.2	-0.5 ± 1.0	-0.7 ± 1.2	-0.4 ± 1.1	-0.1 ± 1.0	<0.001
Animal foods, servings/day						
Animal fat	0.4 ± 0.8	0.4 ± 0.6	0.2 ± 0.5	0.2 ± 0.6	0.1 ± 0.5	<0.001
Daily	0.5 ± 1.7	0.5 ± 1.7	0.0 ± 1.4	-0.1 ± 1.8	-0.2 ± 1.8	<0.001
Eggs	0.2 ± 0.3	0.1 ± 0.3	0.1 ± 0.3	0.1 ± 0.5	0.0 ± 0.3	<0.001
Fish and seafood	0.0 ± 0.2	0.0 ± 0.2	0.0 ± 0.2	0.0 ± 0.2	0.0 ± 0.2	0.17
Meat	0.0 ± 0.6	-0.1 ± 0.7	0.0 ± 0.6	-0.1 ± 0.7	-0.1 ± 0.7	0.032
Miscellaneous animal-based foods	-0.2 ± 1.3	-0.2 ± 1.9	-0.1 ± 0.8	-0.3 ± 1.5	-0.4 ± 1.3	0.10
Food groups, servings/day						
Healthy plant foods	-0.5 ± 4.4	1.2 ± 3.9	2.0 ± 4.6	2.1 ± 3.4	3.3 ± 3.6	<0.001
Less healthy plant foods	-3.3 ± 2.5	-2.4 ± 2.0	-2.2 ± 2.1	-1.6 ± 2.1	-0.9 ± 2.0	<0.001

Supplementary data

Animal foods	0.9 ± 2.5	0.7 ± 2.6	0.2 ± 1.9	-0.2 ± 2.8	-0.6 ± 2.7	<0.001
**Healthful plant-based diet index (hPDI)**						
n	174	174	146	181	156	
Median (range)	-9 (-20, -6)	-3 (-5, -2)	0 (-1, 1)	4 (2, 6)	10 (7, 24)	
Healthy plant foods, servings/day						
Whole grains	-0.5 ± 1.2	-0.3 ± 1.2	-0.2 ± 1.2	0.1 ± 1.3	0.1 ± 1.4	<0.001
Fruits	0.0 ± 1.0	0.2 ± 1.1	0.2 ± 0.9	0.4 ± 1.2	0.6 ± 1.1	<0.001
Vegetables	-0.2 ± 2.1	0.5 ± 1.8	0.6 ± 2.0	0.4 ± 1.6	1.0 ± 2.3	<0.001
Nuts	0.5 ± 0.8	0.6 ± 0.8	0.5 ± 0.8	0.7 ± 1.2	0.9 ± 1.2	<0.001
Legumes	-0.1 ± 0.4	0.1 ± 0.6	0.1 ± 0.5	0.1 ± 0.5	0.2 ± 0.7	<0.001
Vegetable oil	0.2 ± 0.8	0.3 ± 0.5	0.4 ± 0.7	0.3 ± 0.5	0.5 ± 0.7	<0.001
Tea and coffee	-0.1 ± 1.8	-0.1 ± 1.7	-0.1 ± 1.7	0.1 ± 1.8	0.3 ± 1.6	0.010
Less healthy plant foods, servings/day						
Fruit juices	-0.1 ± 0.7	-0.2 ± 0.7	-0.4 ± 0.7	-0.5 ± 0.8	-0.4 ± 0.6	<0.001
Refined grains	-0.3 ± 1.1	-0.6 ± 1.2	-0.6 ± 1.0	-1.1 ± 1.3	-1.5 ± 1.2	<0.001
Potatoes	0.0 ± 0.3	-0.1 ± 0.3	-0.2 ± 0.3	-0.2 ± 0.4	-0.3 ± 0.3	<0.001
Sugar-sweetened beverages	-0.1 ± 0.7	-0.2 ± 0.6	-0.2 ± 0.7	-0.3 ± 0.6	-0.4 ± 0.9	<0.001
Sweets and desserts	-0.2 ± 1.0	-0.5 ± 1.2	-0.5 ± 1.0	-0.6 ± 1.1	-0.9 ± 1.2	<0.001
Animal foods, servings/day						
Animal fat	0.4 ± 0.5	0.3 ± 0.5	0.2 ± 0.7	0.2 ± 0.7	0.1 ± 0.5	<0.001
Daily	0.5 ± 1.5	0.2 ± 1.7	0.2 ± 1.7	0.0 ± 1.7	-0.3 ± 1.8	<0.001
Eggs	0.1 ± 0.4	0.2 ± 0.3	0.1 ± 0.3	0.1 ± 0.3	0.0 ± 0.4	0.002
Fish and seafood	0.0 ± 0.2	0.0 ± 0.2	0.0 ± 0.2	0.0 ± 0.2	0.0 ± 0.3	0.41
Meat	0.1 ± 0.7	0.0 ± 0.7	-0.2 ± 0.6	-0.1 ± 0.6	-0.3 ± 0.7	<0.001
Miscellaneous animal-based foods	-0.3 ± 2.0	-0.1 ± 0.7	-0.1 ± 0.9	-0.3 ± 1.7	-0.3 ± 1.2	0.21
Food groups, servings/day						
Healthy plant foods	-0.2 ± 4.3	1.3 ± 3.9	1.5 ± 4.0	2.2 ± 4.0	3.6 ± 4.2	<0.001
Less healthy plant foods	-0.7 ± 2.1	-1.5 ± 2.0	-1.9 ± 1.9	-2.6 ± 2.0	-3.6 ± 2.4	<0.001
Animal foods	1.0 ± 2.6	0.5 ± 2.1	0.4 ± 2.2	-0.1 ± 2.7	-0.8 ± 2.7	<0.001

Supplementary data

Unhealthful plant-based diet index (uPDI)						
n	157	174	148	192	160	
Median (range)	-17 (-31, -14)	-11 (-13, -9)	-7 (-8, -6)	-3 (-5, -1)	3 (0, 20)	
Healthy plant foods, servings/day						
Whole grains	0.4 ± 1.4	0.1 ± 1.1	-0.3 ± 1.3	-0.3 ± 1.3	-0.6 ± 1.1	<0.001
Fruits	0.8 ± 1.0	0.5 ± 1.1	0.3 ± 1.1	0.2 ± 1.0	-0.2 ± 1.0	<0.001
Vegetables	1.3 ± 1.9	0.6 ± 1.9	0.3 ± 1.9	0.5 ± 2.0	-0.5 ± 2.0	<0.001
Nuts	1.0 ± 1.4	0.7 ± 0.7	0.5 ± 0.8	0.6 ± 0.9	0.3 ± 0.9	<0.001
Legumes	0.2 ± 0.6	0.1 ± 0.4	0.1 ± 0.6	0.1 ± 0.6	-0.1 ± 0.4	<0.001
Vegetable oil	0.5 ± 0.6	0.5 ± 0.6	0.4 ± 0.6	0.3 ± 0.6	0.1 ± 0.7	<0.001
Tea and coffee	0.5 ± 2.0	0.1 ± 1.8	0.1 ± 1.6	-0.1 ± 1.6	-0.5 ± 1.6	<0.001
Less healthy plant foods, servings/day						
Fruit juices	-0.4 ± 0.6	-0.4 ± 0.8	-0.3 ± 0.7	-0.3 ± 0.7	-0.2 ± 0.8	0.004
Refined grains	-1.2 ± 1.4	-1.0 ± 1.3	-0.9 ± 1.2	-0.7 ± 1.2	-0.3 ± 1.0	<0.001
Potatoes	-0.2 ± 0.4	-0.2 ± 0.4	-0.2 ± 0.3	-0.1 ± 0.3	-0.1 ± 0.3	<0.001
Sugar-sweetened beverages	-0.4 ± 1.0	-0.3 ± 0.8	-0.2 ± 0.6	-0.2 ± 0.6	-0.1 ± 0.5	<0.001
Sweets and desserts	-0.8 ± 1.4	-0.5 ± 0.9	-0.5 ± 1.1	-0.5 ± 1.0	-0.4 ± 1.1	<0.001
Animal foods, servings/day						
Animal fat	0.3 ± 0.5	0.4 ± 0.8	0.3 ± 0.6	0.1 ± 0.5	0.2 ± 0.5	<0.001
Daily	0.6 ± 1.6	0.4 ± 1.7	0.1 ± 1.6	-0.1 ± 1.6	-0.3 ± 1.9	<0.001
Eggs	0.2 ± 0.4	0.1 ± 0.3	0.1 ± 0.2	0.1 ± 0.3	0.0 ± 0.4	<0.001
Fish and seafood	0.1 ± 0.2	0.1 ± 0.3	0.0 ± 0.2	0.0 ± 0.2	-0.1 ± 0.2	<0.001
Meat	0.1 ± 0.6	-0.1 ± 0.6	-0.1 ± 0.7	-0.2 ± 0.6	-0.2 ± 0.8	<0.001
Miscellaneous animal-based foods	-0.2 ± 1.2	-0.1 ± 0.7	-0.1 ± 1.0	-0.3 ± 1.4	-0.4 ± 2.3	0.035
Food groups, servings/day						
Healthy plant foods	4.6 ± 3.9	2.6 ± 3.6	1.3 ± 3.6	1.3 ± 3.7	-1.5 ± 3.9	<0.001
Less healthy plant foods	-3.0 ± 2.7	-2.5 ± 2.1	-2.0 ± 2.1	-1.7 ± 2.0	-1.1 ± 2.2	<0.001
Animal foods	1.2 ± 2.4	0.8 ± 2.2	0.2 ± 2.2	-0.4 ± 2.4	-0.8 ± 3.0	<0.001

Supplementary data

[1]Values are means ± SDs changes from baseline (1995) to follow-up (2011). Food group intakes are expressed as servings/day. P is calculated based on the univariate general linear regression model using each index score change as a continuous variable.
n, the number of observations.

Supplementary data

Supplemental Table 2 Cross-sectional associations of plasma adiposity-associated biomarker concentrations with quintiles of overall plant-based diet index (PDI) in 1995 and 2011 in the Nurses' Health study II [1]

	Quintile 1	Quintile 2	Quintile 3	Quintile 4	Quintile 5	% difference/10-point changes (95%CIs)	*P*-trend
Baseline, *n*	212	206	135	155	123		
Follow-up, *n*	112	173	147	215	184		
PDI, median (range)	49 (30-51)	54 (52-55)	57 (56-58)	61 (59-62)	65 (63-78)		
**Adiponectin, ng/mL**							
Multivariable[2]	7.1 (6.7, 7.5)	7.1 (6.7, 7.6)	7.3 (6.9, 7.7)	7.3 (6.9, 7.7)	7.3 (6.8, 7.7)	1.8 (-1.2, 4.9)	0.25
Multivariable+ BMI	7.0 (6.7, 7.4)	7.0 (6.7, 7.4)	7.1 (6.7, 7.6)	7.2 (6.8, 7.6)	7.1 (6.7, 7.5)	1.1 (-1.8, 4.1)	0.47
**Leptin, ng/mL**							
Multivariable	18.8 (16.9, 20.9)	18.3 (16.5, 20.3)	17.0 (15.2, 18.9)	17.8 (16.1, 19.8)	18.0 (16.1, 20.1)	-3.1 (-8.5, 2.7)	0.29
Multivariable+ BMI	18.2 (16.7, 19.8)	18.1 (16.6, 19.6)	17.3 (15.9, 18.8)	17.6 (16.2, 19.2)	17.8 (16.3, 19.4)	-1.7 (-6.4, 3.3)	0.50
**sOB-R, ng/mL**							
Multivariable	23.8 (22.9, 24.7)	24.2 (23.3, 25.1)	24.8 (23.9, 25.7)	24.2 (23.3, 25.1)	24.6 (23.7, 25.5)	1.8 (-0.2, 3.8)	0.08
Multivariable+ BMI	23.8 (23.0, 24.6)	24.1 (23.3, 24.9)	24.5 (23.6, 25.3)	24.0 (23.2, 24.9)	24.2 (23.4, 25.1)	0.9 (-1.0, 2.9)	0.34
**Free leptin index**[3]							
Multivariable	0.8 (0.7, 0.9)	0.8 (0.7, 0.9)	0.7 (0.6, 0.8)	0.7 (0.6, 0.8)	0.7 (0.6, 0.8)	-5.6 (-12.0, 1.2)	0.11
Multivariable+ BMI	0.8 (0.7, 0.8)	0.8 (0.7, 0.8)	0.7 (0.6, 0.8)	0.7 (0.7, 0.8)	0.7 (0.7, 0.8)	-3.2 (-8.8, 2.8)	0.29
**Insulin, uU//mL**							
Multivariable	6.1 (5.5, 6.7)	5.4 (4.9, 6.0)	5.4 (4.9, 6.0)	5.3 (4.8, 5.9)	5.2 (4.7, 5.8)	-8.1 (-13.6, -2.3)	0.007
Multivariable+ BMI	6.0 (5.4, 6.6)	5.4 (4.9, 5.9)	5.6 (5.0, 6.1)	5.4 (4.9, 6.0)	5.5 (5.0, 6.1)	-4.1 (-9.4, 1.5)	0.15
**RBP-4, µg/mL**							
Multivariable	36.7 (35.5, 37.8)	36.7 (35.6, 37.9)	35.5 (34.4, 36.6)	35.6 (34.5, 36.8)	36.0 (34.8, 37.1)	-1.8 (-3.6, 0.0)	0.05
Multivariable+ BMI	36.5 (35.4, 37.7)	36.6 (35.5, 37.8)	35.5 (34.4, 36.6)	35.6 (34.5, 36.7)	36.0 (34.9, 37.2)	-1.5 (-3.3, 0.3)	0.11
**hsCRP, mg/L**							
Multivariable	1.11 (0.92, 1.33)	1.08 (0.90, 1.28)	1.02 (0.85, 1.22)	0.98 (0.82, 1.17)	0.90 (0.75, 1.09)	-11.8 (-20.5, -2.2)	0.018
Multivariable+ BMI	1.08 (0.91, 1.28)	1.08 (0.92, 1.28)	1.06 (0.90, 1.26)	1.00 (0.84, 1.18)	0.96 (0.81, 1.14)	-7.5 (-16.1, 2.0)	0.12
**IL-6, pg/mL**							
Multivariable	0.86 (0.78, 0.94)	0.89 (0.81, 0.97)	0.92 (0.84, 1.01)	0.92 (0.84, 1.01)	0.88 (0.80, 0.96)	2.4 (-3.1, 8.2)	0.40
Multivariable+ BMI	0.84 (0.76, 0.91)	0.88 (0.80, 0.96)	0.93 (0.85, 1.02)	0.92 (0.85, 1.01)	0.90 (0.82, 0.99)	5.5 (0.0, 11.3)	0.05

[1]Values are least squares geometric means (95% CIs) from linear mixed model to account for within-person correlation between the two blood measurement time points.

[2]Adjusted for age, time period, total energy intake, alcohol intake, smoking status, physical activity, menopausal status, postmenopausal hormone use, hypertension, and hypercholesterolemia.

[3]Free leptin index is defined as the ratio of leptin to soluble leptin receptor.

BMI, body mass index; hsCRP, high-sensitivity C-reactive protein; IL-6, interleukin-6; *n*, the number of observations; PDI, overall plant-based diet index; RBP-4, retinol-binding protein-4; sOB-R, soluble leptin receptor.

Supplemental Table 3 Percentage changes in plasma adiposity-associated biomarker concentrations across quintiles of overall plant-based diet index (PDI) change between 1995 and 2011 in the Nurses' Health study II [1]

	Quintile1	Quintile 2	Quintile 3	Quintile 4	Quintile 5	*P*-trend
n	161	162	197	131	180	
Median (range)	-6 (-18, -4)	-1.5 (-3, 0)	3 (1, 4)	6 (5, 7)	10 (8, 27)	
**Adiponectin**						
Multivariable[2]	0.5 (-7.7, 9.5)	5.9 (-2.7, 15.3)	1.0 (-6.9, 9.6)	1.0 (-7.3, 10.0)	0.5 (-8.0, 9.8)	0.55
Multivariable+ Wt change	3.2 (-4.9, 12.0)	8.6 (0.1, 17.7)	2.3 (-5.4, 10.5)	4.1 (-4.0, 13.0)	3.1 (-5.2, 12.1)	0.52
**Leptin**						
Multivariable	39.2 (18.2, 64.0)	24.8 (6.1, 46.8)	38.9 (18.8, 62.3)	43.7 (22.0, 69.2)	34.0 (13.2, 58.7)	0.71
Multivariable+ Wt change	29.0 (12.5, 48.0)	17.1 (2.2, 34.1)	32.8 (16.6, 51.4)	30.8 (14.0, 50.1)	22.7 (6.5, 41.3)	0.88
**sOB-R**						
Multivariable	-3.4 (-8.6, 2.2)	-1.4 (-6.7, 4.2)	-2.3 (-7.4, 3.0)	-4.3 (-9.5, 1.2)	-2.5 (-8.0, 3.2)	0.91
Multivariable+ Wt change	-2.0 (-7.1, 3.4)	-0.1 (-5.3, 5.3)	-1.7 (-6.5, 3.5)	-2.5 (-7.6, 2.8)	-0.9 (-6.2, 4.7)	0.99
**Free leptin index**[3]						
Multivariable	43.3 (17.2, 75.2)	29.5 (6.2, 58.0)	40.7 (16.2, 70.4)	50.9 (23.5, 84.5)	38.5 (12.7, 70.3)	0.73
Multivariable+ Wt change	32.0 (11.0, 56.9)	21.4 (2.3, 44.0)	34.9 (14.4, 58.9)	37.0 (15.2, 62.9)	26.4 (5.9, 51.0)	0.83
**Insulin**						
Multivariable	23.1 (2.0, 48.5)	28.6 (6.9, 54.6)	29.0 (8.0, 54.1)	23.1 (2.1, 48.4)	18.6 (-2.0, 43.5)	0.57
Multivariable+ Wt change	18.7 (-0.7, 41.9)	24.8 (4.7, 48.7)	26.4 (6.8, 49.7)	17.3 (-1.9, 40.2)	13.3 (-5.5, 35.9)	0.45
**RBP-4**						
Multivariable	12.6 (6.6, 18.9)	11.0 (5.1, 17.1)	14.1 (8.3, 20.3)	9.4 (3.6, 15.6)	9.3 (3.4, 15.7)	0.29
Multivariable+ Wt change	12.1 (6.1, 18.4)	10.6 (4.8, 16.7)	13.9 (8.1, 19.9)	8.9 (3.0, 15.0)	8.8 (2.8, 15.1)	0.27
**hsCRP**						
Multivariable	9.0 (-17.6, 44.3)	3.9 (-21.2, 37.0)	0.6 (-23.0, 31.5)	-3.4 (-27.1, 27.9)	12.7 (-15.5, 50.4)	0.98
Multivariable+ Wt change	2.6 (-21.5, 34.1)	-2.0 (-24.8, 27.7)	-2.2 (-24.2, 26.2)	-10.7 (-31.7, 16.8)	4.6 (-20.6, 37.7)	0.92
**IL-6**						
Multivariable	12.7 (-4.5, 33.1)	9.0 (-7.5, 28.5)	10.6 (-5.5, 29.6)	19.7 (1.3, 41.4)	19.3 (0.6, 41.6)	0.27
Multivariable+ Wt change	11.4 (-5.6, 31.5)	7.7 (-8.7, 27.0)	10.3 (-5.8, 29.1)	17.8 (-0.4, 39.2)	17.8 (-0.7, 39.8)	0.27

[1]Values are adjusted least squares percent changes (95% CIs) in plasma biomarker concentrations from general linear model.
[2]Adjusted for age at the first blood measurement, changes in total energy intake, alcohol intake, smoking status, physical activity, menopausal status, postmenopausal hormone use, hypertension, hypercholesterolemia, baseline BMI, baseline overall plant-based diet index, and baseline corresponding biomarker concentrations.
[3]Free leptin index is defined as the ratio of leptin to soluble leptin receptor.
hsCRP, high-sensitivity C-reactive protein; IL-6, interleukin-6; *n*, the number of observations; PDI, overall plant-based diet index; RBP-4, retinol-binding protein-4; sOB-R, soluble leptin receptor; Wt, weight.
